# Epidemic Plasmid Carrying *bla*_CTX-M-15_ in *Klebsiella penumoniae* in China

**DOI:** 10.1371/journal.pone.0052222

**Published:** 2013-01-29

**Authors:** Chao Zhuo, Xiao-qiang Li, Zhi-yong Zong, Nan-Shan Zhong

**Affiliations:** 1 State Key Laboratory of Respiratory Diseases, Guangzhou Medical College, Guangzhou, China; 2 Department of Infectious Diseases, West China Hospital, Sichuan University, Chengdu, China; Universitätsklinikum Hamburg-Eppendorf, Germany

## Abstract

**Objective:**

To investigate the local epidemiology of *Klebsiella penumoniae* carrying *bla*_CTX-M-15_ in southern China and to characterize the genetic environment of *bla*_CTX-M-15_.

**Methods:**

PCR and DNA sequencing were used to detect and characterize the genetic contexts of *bla*_CTX-M-15_. The clonal relatedness of isolates carrying *bla*_CTX-M-15_ was determined by pulse-field gel electrophoresis. Conjugative plasmids carrying *bla*_CTX-M-15_ were obtained by mating and were further subject to restriction analysis and replicon typing.

**Results:**

A total of 47**CTX-M-15 ESBL-producing isolates of *K. pneumoniae* were collected from nine hospitals in China from October 2007 to October 2008. Isolates were clustered into various clonal groups. The local spread of *bla*_CTX-M-15_ was mainly mediated by one major conjugative plasmid as determined by S1-PFGE and restriction analysis. A 90-kb plasmid belonging to incompatible group FII was the major carrier of *bla*_CTX-M-15_ in *K. pneumoniae*. Except *bla*_TEM-1_, the resistance genes such as *bla*_SHV_, *bla*_DHA-1_, *bla*_OXA-1_, *qnrB*, *qnrS*, *aac(3)-II*, and *aac(6′)-Ib* were not found in the plasmid. In the comparing of conjugative gene sequence, it is 100% identical with the plasmid pKF3–94, which was found in *K. pneumonia* from Zhejiang province of china previously.

**Conclusions:**

*bla*_CTX-M-15_ was prevalent in *K. pneumonia* of southern China. The dissemination of *bla*_CTX-M-15_ appeared to be due to the horizontal transfer of a 90-kb epidemic plasmid.

## Introduction

CTX-M enzymes have emerged as the predominant type of extended-spectrum β-lactamases (ESBL) produced by clinical isolates of *Enterobacteriaceae* in the world [1]. At present, more than 90 CTX-M variants have been designated (http://www.lahey.org/Studies/other.asp), of which CTX-M-15 is the most prevalent variant globally [2]. The global spread of *bla*_CTX-M-15_ is largely due to *Escherichia coli* of sequence type (ST) 131 and IncFII plasmids [3], [4]. In China, *bla*_CTX-M-14_ has been the dominant *bla*_CTX-M_ variant, especially in *E. coli* [5], [6]. A recent small-scale investigation shown that 64.7% (11/17) *E. coli* isolates and 27.3% (3/11) *K. pneumoniae* isolates carried *bla*_CTX-M-14_ [7]. Interestingly, 64.3% of the *bla*_CTX-M-14_ genes were mainly located on an IncF plasmid in the 14 *bla* ^CTX-M-14^-positive isolates, which indicate the IncF plasmids plays a main role in dissemination of *bla* ^CTX-M-14^ among the *E. coli* isolates in China [7].

The resistant rate of ceftazidime has increased sharply in both *E. coli* and *K. pneumoniae* recently in China [8]. However, the data about *bla*_CTX-M-15_ in *Klebsiella pneumoniae* is scarce, especially the clonal relatedness of the plasmids in *K. pneumoniae* isolates carrying *bla*_CTX-M-15_ in China. Owing to CTX-M-15 enzyme hydrolyse ceftazidime at a higher rate than cefotaxime, it was speculated that *bla*_CTX-M-15_ gene may be emerging and spreading in China. Our previous study has also shown that *bla*_CTX-M-15_ had emerged as the more common type of *bla*_CTX-M_ genes in *K. pneumoniae* in Guangzhou during 2007 to 2008, and revealed that 28.3% and 26.1% ESBL-producing *K. pneumoniae* isolates carried *bla*_CTX-M-14_ and *bla*_CTX-M-15,_ respectively [9]. The mechanisms responsible for the high prevalence of *bla*_CTX-M-15_ in *K. pneumoniae* occurred in the period remained undetermined. Therefore, all of the 47 *K. pneumoniae* isolates carrying *bla*_CTX-M-15_ from our previous study were studied for clonal relatedness and were also subjected to plasmid analysis including replicon typing and restriction analysis. This study show that *bla*_CTX-M-15_ was prevalent in *K. pneumoniae* of southern China and the dissemination of *bla*_CTX-M-15_ appeared to be due to the spread of a 90-kb epidemic plasmid.

## Materials and Methods

### Clinical Isolates

Clinical *K. pneumoniae* isolates carrying *bla*_CTX-M-15_ identified in our pervious study [9] were collected from nine hospitals, as part of the MOH National Antimicrobial Resistant Investigation net (Mohnarin) Program from October 2007 to October 2008. Briefly, Nine hospitals are scattered geographically over the three cities in southern China, Hospital A, B, C, D, E are located in the city I, and hospital F, G, H in the city II, and hospital K in the city III. Hospital A, B, C are close to each other, and the nearest distance between two hospitals is about 1 km, the city II and city III are far from the city I, and the distance is about 100 km and 300 km, respectively. Sometimes, the same patients may walk through the hospitals in one city for treatment. All the 181 isolates *K. pneumoniae* producing ESBLs were screened by PCR with special primers for all known CTX-M type encoding gene [9]. Purified PCR products were directly sequenced from both ends or cloned in pMD18-T and then sequenced. The DNA sequences and deduced amino acid sequences were compared to genes in GenBank or the β-lactamases classification system (www.lahey.org/studies/webt.html) to conform the subtypes of β-lactamase genes. The species identification for all isolates was performed using the VITEK-2 (bioMérieux, Marcy l’Etoile, France) automated microbiological analyzing system.

### Antimicrobial Susceptibility Testing

Antimicrobial susceptibility testing of isolates and their transconjugants carrying *bla*_CTX-M-15_ was performed using the microdilution method according to Clinical and Laboratory Standards Institute (CLSI) guidelines [10]. Antimicrobial agents tested were ampicillin, ampicillin-sulbactam, piperacillin, piperacillin-tazobactam, cefoperazone-sulbactam, cefoxitin, cefotaxime, ceftazidime, cefepime, imipenem, aztreonam, amikacin and ciprofloxacin. All of the agents were provided by the Chinese National Institute for the Control of Pharmaceutical and Biological Products, except for cefoperazone-sulbactam, which was obtained from Pfizer (New York, NY). *E. coli* ATCC 25922 was used as the control strain. Isolates were classified as susceptible or resistant according to the interpretative standards recommended by CLSI.

### Clonal Relatedness of Isolates Carrying *bal*_CTX-M-15_

Pulsed-field gel electrophoresis (PFGE) analysis of *Xba*I-digested genomic DNA was performed to determine the genetic relatedness of CTX-M-producing *K. pneumoniae* isolates using a CHEF-Mapper XA System (Bio-Rad Laboratories, Hercules, CA, USA) as described by Seifert [11]. The interpreting criteria were described by Tenover [12] combining UPGMA (unweighted pair group method with hierarchic averages) method, Isolates were assigned the same pulsetype if the value of Dice coefficient of similarity was >80% [13].

### Conjugation

Conjugation experiments were performed in broth as described previously [14]. *K. pneumoniae* strains of different clones carrying *bla*_CTX-M-15_ were used as donor strains, while a rifampicin-resistant variant of *E. coli* strain C600, C600 (*Rif* ^r^), was used as the recipient. Transconjugants were selected on MH agar (Oxoid, Basingstoke, UK) supplemented with ceftazidime (2 µg ml^−1^) plus rifampicin (300 µg ml^−1^).

### Plasmid Analysis

Plasmids from transconjugants were prepared using a modified alkaline lysis method [15]. Plasmid DNA was linearized with the S1 enzyme followed by PFGE [16]. The estimation of the plasmid size was compared with the molecular weight marker, *Salmonella braenderup* H9812. Plasmids were restricted with *Eco*RI. Plasmid replicons were determined using the PCR-based replicon typing scheme(PBRT) by using eighteen pairs of primers to perform PCR which recognized F,FIA, FIB, FIC, HI1, HI2, I1-Ic, L/M, N, P, W, T, A/C, K, B/O, X, Y and FII replicons as described by Carattoli [17]. All the primers and targets were listed in Table S2. To further determine the backbone of the plasmid, the genes *traF, traH, traN, traU, traW and trbC*, specific to the backbone of F system and ought to demonstrate the relationship of plasmid [18], were employed to analyze the backbone of the 90-kb plasmid by PCR and sequenced. (Primers listed in Table 1).

**Table 1:** Primers used in this study.

Primer	sequence (5′→3′)	GenBank accession no
blaCTX-M group	F:ATGTGCAGYACCAGTAARGTKATGGC	AY458016
	R:TGGGTRAARTARGTSACCAGAAYCAGCGG	
blaCTX-M-1 group	F:CAGCGCTTTTGCCGTCTAAG	AY458016
	R:GGCCCATGGTTAAAAAATCACTGC	
blaTEM	F: GAGTATTCAACATTTTCGT	AY458016
	R: ACCAATGCTTAATCAGTGA	
blaSHV	F:CGCCGGGTTATTCTTATTTGTCGCG	X98101
	R:TCTTTCCGATGCCGCCGCCAGTCA	
blaOXA	F:CCAAAGACGTGGATG	FJ594766
	R:GTTAAATTCGACCCCAAGTT	
blaDHA-1	F:CTCATCCTCCATAAAACAGC	FJ715937
	R:TTATCTCACACCTTTATTACT	
qnrA	F:AAGGAAGCCGTATGGATATT	AB469045
	R:AGCTAATCCGGCAGCACTAT	
qnrB	F:CGACCTGAGCGGCACTGAAT	FJ233873
	R:TGAGCAACGATGCCTGGTAG	
qnrS	F:ACCTTCACCGCTTGCACATT	FJ418153
	R:CCAGTGCTTCGAGAATCAGT	
aac-(6′)-Ib-cr	F:ATGACTGAGCATGACCTTGC	FJ790516
	R:TTAGGCATCACTGCGTGTTC	
	R:CTCGAATGCCTGGCGTGTTT	
aac(3′)-II	F:ATATCGCGATGCATACGCGG	GQ343184.1
	R:GACGGCCTCTAACCGGAAGG	
traF	F:TGGCAGTGGTATAACGAGA	NC005327
	R:CCATAGGTATCCCTGAAGC	
traH	F:CTATGGTGGCTCCCTGTAT	NC005327
	R:TGTTCTGGTAACGGCTGA	
traN	F:TGTGGTGGTGATGTCTTCTG	NC005327
	R:CAAACCCGATACGCAACT	
traU	F:CCATTGGTTACTGGGAGC	NC005327
	R:GCGTTCTTTAGGCAGGATT	
traW	F:GTATCGGACGCACGGAGA	NC005327
	R:AGTAAACACGGCTGTCCAGAG	
traC	F:AGGGTGCCCTGTATTTTGTGTCGTT	NC005327
	R:TGGCGGCCACTTTCTCCACG	

K = G or T; R = A or G; S = C or G; Y = C or T.

To determine whether some other resistance genes and insertion sequences were co-transferred with *bla*_CTX-M-15_, transconjugants obtained were screened for IS*Ecp1,* IS26, *bla*_TEM_, *bla*_SHV_, *bla*_DHA-1_, *bla*_OXA-1_, *qnrB*, *qnrS*, *aac(3)-II*, and *aac(6')-Ib* by PCR (primers listed in Table 1).

## Results

### Antimicrobial Susceptibility Testing

A total of 47 out of 125 *bla*_CTX-M_ postive strains of *K. pneumoniae* carrying *bla*_CTX-M-15_, were originated from sputum (n = 38), blood (n = 6), abscess (n = 3). Results of the microdilution method showed that both types of ESBL-producing strains had low resistance to imipenem, piperacillin/Tazobactam and cefoperazone/sulbactam, with the resistance rate of 2.1%, 10.6% and 14.9%, respectively. The third-generation cephalosporin and cefepime had an antimicrobial resistance rate ranging from 57.4% to 89.5% (Table S1).

### Clonal Relatedness of Isolates Carrying *bla*_CTX-M-15_

**Figure 1:**
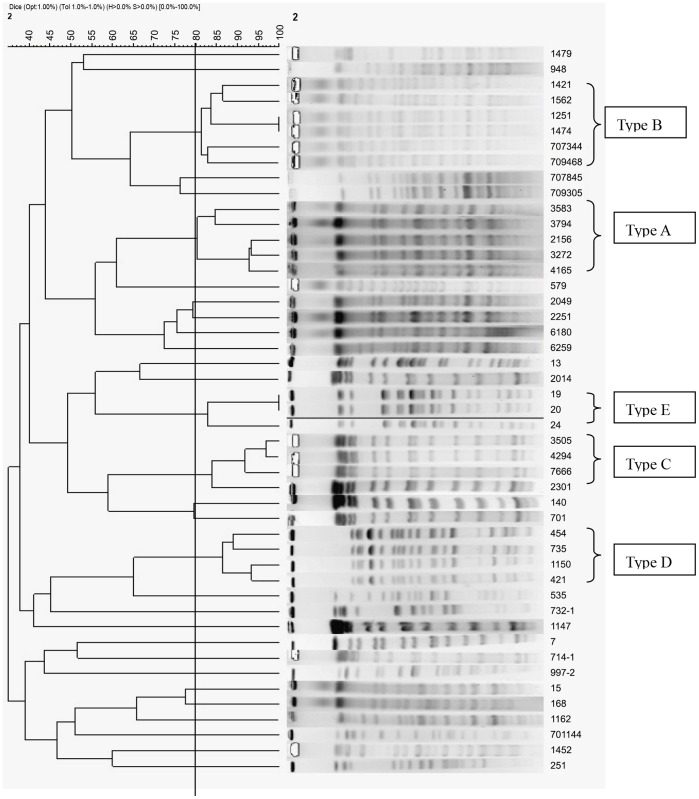
Dendrogram to illustrate the relatedness of 47 CTX-M-15 producing *K. pneumoniae* isolates. The same pulsetype defined by PFGE profiles with ≥80% similarity (UPGMA, Dice; black vertical line) are indicated. 30 pulsotypes were designated.

According to the patterns of PFGE isolates, 30 pulsotypes were designated for 47 *bla*_CTX-M-15-_producing *K. pneumoniae* isolates (Fig. S4). Besides 25 individual pulsotypes, the remaining 22 isolates collected from three hospitals were classified into 5 pulsotypes, designated types A, B, C, D and E (Fig. 1). Five isolates from hospital A belonged to type A, 3 isolates from hospital A and 3 isolates from hospital B belonged to type B, 4 isolates from hospital B belonged to type C, 2 isolates from hospital B and 2 isolates from hospital C belonged to type D, 3 isolates from hospital C belonged to type E. In summary, the small clonal dissemination just occurred in each hospital or near hospital. There is no a predominant clone carrying *bla*_CTX-M-15_ found in this study.

### Antibiotic Resistance Profile of 30 *K. pneumoniae* Isolates and the Transconjugants

For isolates of the same pulsotype, only one was chosen as a representative for further study. Therefore, a total of 30 non-clonal isolates carrying *bla*_CTX-M-15_ were subject to plasmid analyzing. The conjugative plasmids were obtained in all the 30 isolates, and 21 transconjugants containing a single 90-kb conjugative plasmid carrying *bla*_CTX-M-15,_ while 9 transconjugants containing three different size of plasmid (60-kb,90-kb,160-kb) (Fig. S1). The susceptibility characteristics of all transconjugants are shown in Table 2. All the transconjugants were susceptible to imipenem and ciprofloxacin, while almost of them were resistant to ampicillin, piperacillin, aztreonam and cefotaxime. For these above antimicrobial agents, transconjugants shared similar MICs with their corresponding parent strains. In addition, the MIC of ceftazidime for transconjugants, were ranged from 4 to 64 mg/l, with 2–8 folds decreased than the parent strains, respectively. We also observed that 30.0% (9/30) isolates and 83.3% (25/30) transconjugants were susceptible to ceftazidime, according to CLSI breakpoint.

**Table 2:** MICs of antimicrobial agents for *K. pneumoniae* clinical isolates and the transconjugants.

strain	AMP	SAM	PIP	FOX	CTX	CAZ	FEP	CSL	TZP	AZM	IPM	CIP	AMK
K7	≥256	32	≥256	4	≥256	32	64	16	16	64	0.5	1	32
Tr7	≥128	8	≥256	2	32	8	32	2	2	≥128	≤0.25	0.5	0.5
K13	8	4	16	32	8	16	4	16	8	8	0.5	0.5	1
Tr13	8	2	8	2	8	4	2	4	4	8	≤0.25	0.5	≤0.25
K15	≥256	128	≥256	64	≥256	64	64	≥128	16	≥256	0.5	4	64
Tr15*	≥128	8	≥256	32	64	32	32	16	8	64	≤0.25	0.5	32
K20	≥256	64	≥256	4	≥256	16	64	16	16	64	≤0.25	1	8
Tr20	≥128	4	≥256	0.5	16	8	32	4	8	64	≤0.25	0.5	≤0.25
K24	≥256	64	≥256	4	≥256	32	64	32	16	128	≤≤0.25	0.5	8
Tr24	≥128	4	≥256	0.5	64	4	32	8	4	≥128	≤0.25	0.5	1
K140	16	8	16	8	4	16	8	2	2	16	0.5	2	16
Tr140	16	2	16	1	4	2	4	1	1	16	≤0.25	0.5	1
K251	≥256	64	≥256	32	≥256	8	64	16	8	64	≤0.25	1	4
Tr251	≥128	8	≥256	2	≥128	4	32	4	4	64	≤0.25	0.5	0.5
K535	≥256	64	≥256	4	≥256	16	64	16	16	≥256	0.5	0.5	4
Tr535	≥128	16	≥256	0.5	32	4	32	4	1	≥128	≤0.25	0.5	0.5
K579	≥256	8	≥256	≥128	≥256	32	≥128	32	16	≥256	0.5	2	64
Tr579*	≥128	2	≥256	64	≥128	32	64	4	4	≥128	≤0.25	0.5	32
K714-1	≥256	8	≥256	4	≥256	8	64	16	16	64	≤0.25	1	8
Tr714-1	≥128	2	≥256	≤0.25	≥128	8	32	2	4	64	≤0.25	0.5	1
K732-1	≥256	8	≥256	4	≥256	≥128	64	8	4	64	0.5	2	8
Tr732-1	≥128	1	≥256	1	≥128	32	16	4	1	32	0.5	0.5	2
K948	≥256	32	≥256	8	≥256	4	8	8	8	128	0.5	1	16
Tr948	≥128	4	≥256	1	≥128	4	4	4	4	≥128	≤0.25	0.5	2
K997-2	≥256	8	≥256	4	≥256	32	32	4	8	64	≤0.25	0.5	16
Tr997-2	≥128	1	≥256	1	≥128	4	8	2	2	64	≤0.25	0.5	0.5
K1147	≥256	32	≥256	4	≥256	8	≥128	16	16	128	≤0.25	1	8
Tr1147	≥128	8	≥256	0.5	≥128	4	64	16	4	64	≤0.25	0.5	1
K1150	≥256	64	≥256	4	≥256	32	64	64	≥128	64	0.5	2	32
Tr1150	≥128	8	≥256	1	64	8	64	16	16	32	0.5	0.5	0.5
K1162	≥256	4	≥256	4	≥256	4	8	4	2	64	≤0.25	1	64
Tr1162	≥128	0.5	≥256	2	64	4	4	2	0.5	64	≤0.25	0.5	0.5
K1452	≥256	128	≥256	8	≥256	≥128	64	≥128	64	≥256	0.5	≥8	64
Tr1452	≥128	64	≥256	2	≥128	32	16	16	16	≥128	≤0.25	0.5	16
K1474	≥256	64	≥256	2	≥256	8	64	64	≥128	≥256	≤0.25	0.5	8
Tr1474	≥128	2	≥256	0.5	≥128	4	8	16	32	≥128	≤0.25	0.5	0.5
K1479	≥256	64	≥256	8	≥256	16	16	32	64	64	≤.25	2	2
Tr1479	≥128	4	≥256	0.5	32	8	4	8	8	64	≤0.25	0.5	0.5
K1562	≥256	64	≥256	4	≥256	16	64	32	32	≥256	≤0.25	0.5	8
Tr1562	≥128	2	≥256	1	64	8	64	8	4	≥128	≤0.25	0.5	0.5
K2014	≥256	64	≥256	4	≥256	≥128	64	32	32	≥256	≤0.25	4	32
Tr2014	≥128	2	≥256	0.5	64	16	16	4	4	64	≤0.25	0.5	0.5
K2251	≥256	128	≥256	4	≥256	8	64	16	16	64	≤0.25	0.5	2
Tr2251	≥128	64	≥256	1	≥128	4	16	16	8	64	≤0.25	0.5	0.5
K2301	≥256	8	≥256	8	≥256	16	8	8	4	≥256	0.5	≥8	16
Tr2301	≥128	4	≥256	2	≥128	2	2	4	2	≥128	≤0.25	0.5	0.5
k3272	≥256	8	≥256	64	≥256	64	64	4	16	64	≤0.25	0.5	0.5
Tr3272	64	4	≥256	4	64	8	16	4	4	64	≤0.25	0.5	0.5
K4294	≥256	64	≥256	8	≥256	8	8	4	4	64	1	4	64
Tr4294	64	8	≥256	2	≥128	4	4	4	1	64	≤0.25	0.5	0.5
K6180	≥256	64	≥256	4	≥256	32	16	16	16	≥256	≤0.25	0.5	1
Tr6180	≥128	16	≥256	0.5	64	8	4	8	4	≥128	≤0.25	0.5	0.5
K6259	≥256	8	≥256	32	≥256	32	64	8	8	64	1	4	64
Tr6259*	≥128	2	≥256	4	≥128	8	8	2	2	64	0.5	0.5	0.5
K701144	≥256	16	≥256	8	≥256	64	64	8	4	≥256	≤0.25	0.5	4
Tr701144	≥128	2	≥256	1	≥128	8	64	4	4	64	≤0.25	0.5	0.5
K707344	≥256	16	≥256	4	≥256	≥128	≥128	16	32	≥256	1	4	16
Tr707344	≥128	4	≥256	0.5	64	8	64	4	4	64	≤0.25	0.5	0.5
K709305	≥256	128	≥256	4	≥256	8	4	64	64	128	0.5	≥8	8
Tr709305	≥128	16	≥256	0.5	64	4	2	8	4	≥128	≤0.25	0.5	1
RecipientC600 (*Rif* ^r^)	≤2	≤2	≤0.25	0.5	0.5	≤0.25	≤0.25	2	0.25/4	≤0.25	≤0.25	≤0.25	≤0.25

Note: K type: clinical isolates of *K. pneumoniae.* Tr type: transconjugants.

* the transconjugants containing three different size of plasmid (60 kb,90 kb,160 kb) AMP:Ampicillin SAM:Ampicillin/Sulbactam PIP:Piperacillin FOX:Cefoxitin CTX:Cefotaxime CAZ:Ceftazidime FEP:Cefepime CSL:Cefoperazone/Sulbactam TZP:Piperacillin/Tazobactam AZM:Aztreonam IPM:Imipenem CIP:Ciprofloxacin AMK:Amikacin.

### Plasmids Carrying *bla*_CTX-M-15_

**Figure 2:**
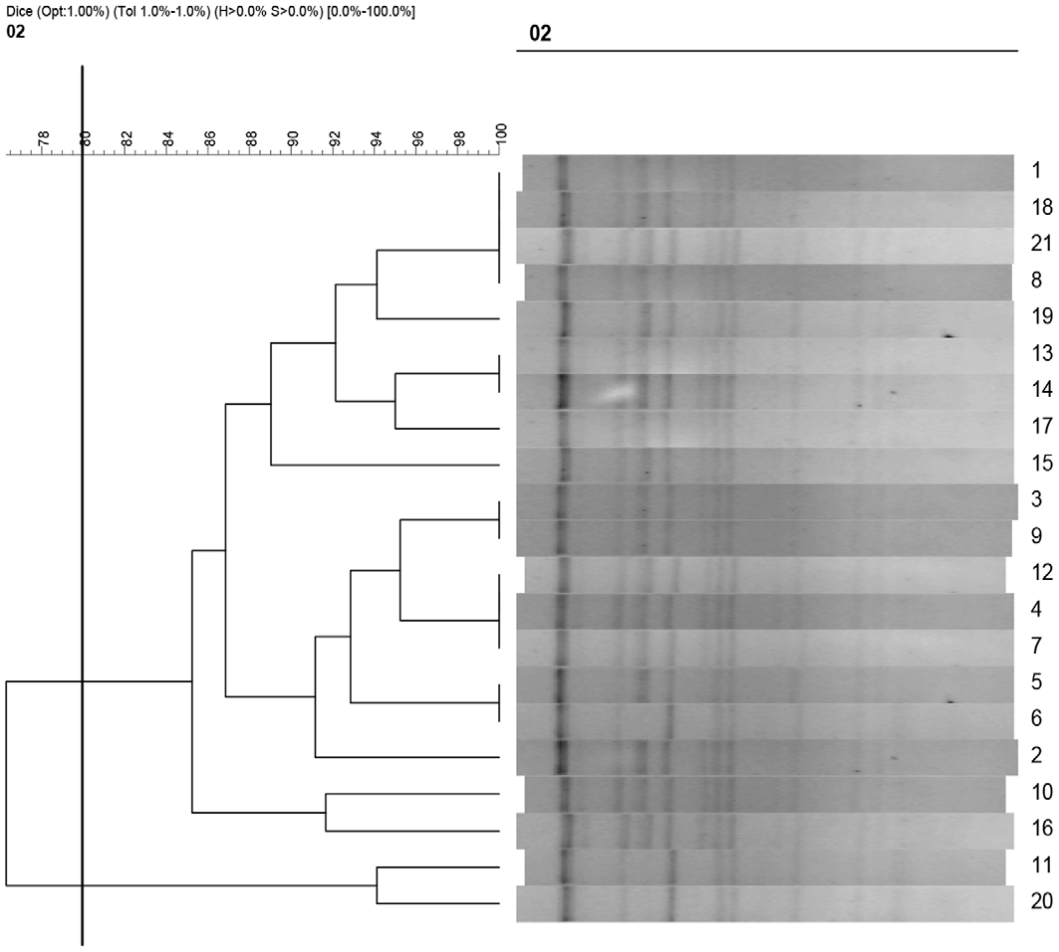
Dendrogram to illustrate the similarity of 90-kb plasmid digested by the EcoRI. The similar patterns were defined with ≥80% similarity (UPGMA, Dice; black vertical line). 21 transconjugants containing a single 90-kb conjugative plasmid carrying *bla*_CTX-M-15_ were analyzed.

To determine the role of plasmid in dissemination of *bla* ^CTX-M-15,^ the single 90-kb conjugative plasmids were subject to analyzed. Firstly, all the 90-kb plasmids were confirmed to be the IncFII group by PCR-based replicon typing (PBRT). Restriction analysis revealed that the twenty-one 90-kb plasmids restricted with *Eco*RI had highly similar patterns (Figure 2). The sequences of *traF, traH, traN, traU, traW* and *trbC* in all the 90-kb plasmids were 100% identical to the plasmid pKF3-94 which is an epidemic plasmid carried *bla*_CTX-M-15_ in Zhejiang province of China [18]. Meanwhile, these sequences were found to have low identities to those of plasmid pC15-1a though both were in similar size. Besides, we also found that the length of EcoRI-EcoRI fragment of pKF3-94 mated with some restriction fingerprints of the epidemic plasmid theoretically, as shown in Table S3, there are 13 restriction fragments digested by *Eco*RI in pKF3-94 with the similar length of fragments of the restricted plasmids.

To demonstrate that the *bla*_CTX-M-_15 is located on the 90-kb plasmid of the remaining 9 transconjugants which contains multi-plasmid (60-kb, 90-kb, 160-kb) (Fig. S2), the 90-kb plasmid DNA was acquired by plasmid electrophoresis and gel extraction, and then it was used as a template, *bla*_CTX-M-15_ was amplified by PCR and sequenced. Together, these results indicated that all the 90-kb plasmid harbored *bla*_CTX-M-15_.

### Genetic Environment of *bla*_CTX-M-15_

To compare the resistance region between the 90-kb plasmid and other epidemic plasmids carrying *bla*_CTX-M-15_, the common elements involved in the mobilization and expression of *bla*_CTX-M-15_ gene were analyzed. The insertion of IS*Ecp1* was observed at 48 bp upstream of the start codon of all the 47 CTX-M-15 group genes by DNA sequence analysis. IS*26*, related to the transmission of β-lactamase genes such as DHA-1, CFE-1, ACC-1 and SHV-2a, was usually found in the IncFII plasmid [19]–[20]. However, PCR amplification with primers specific for the *tnpA* genes of IS*26* was negative for all 47 CTX-M-15 groups. In addition, *bla*_TEM-1_ was detected in all the 90-kb plasmid of transconjugants by sequenced. Other resistance genes such as *bla*_SHV_, *bla*_DHA-1_, *bla*_OXA-1_, *qnrB*, *qnrS*, *aac(3)-II*, and *aac(6′)-Ib* were not found in the 90-kb plasmids consistent with the MIC data.

## Discussion

Here, we show that *K. pneumoniae* carrying *bla*_CTX-M-15_ have emerged in southern China. The local spread of *bla*_CTX-M-15_ in *K. pneumoniae* was not mainly due to one or several dominant clones as various pulsotypes were identified in this study, though there were some medical tourism might occurred among the nine hospitals in the study period, unlike the situation seen in *E. coli* ST131[21].

In contrast to the diversity of clonal relationships, many local isolates harbored a 90-kb IncFII plasmid carrying *bla*_CTX-M-15_, suggesting that this plasmid appeared to be a major vehicle mediating the local dissemination of *bla*_CTX-M-15_ in *K. pneumoniae*. Indeed, previous reports [1] suggested that plasmid is one major factor responsible for the worldwide spread of *bla*_CTX-M-15_. For example, in *E. coli*, many plasmids carrying *bla*_CTX-M-15_ found in France, Tunis, Bangui and India [22]–[23], shared common features with pC15-1a from Canada [3]. Furthermore, emergence of *K. pneumoniae* isolates producing CTX-M-15 were also found in European countries and *bla*_CTX-M-15_ transfer were mediated by IncFII-related plasmids with different sizes among part of them [24]. In this study, non-clonal isolates of *K. pneumoniae* from different hospitals had the same plasmid carrying *bla*_CTX-M-15_. To our best knowledge, this is the first evidence of an epidemic plasmid carrying *bla*_CTX-M-15_ in *K. pneumoniae*.

IncFII-related plasmids are narrow-host range plasmids that are frequently involved in the worldwide dissemination of the *bla*_CTX-M-15_ gene [1]. However, unlike plasmids belonging to other incompatibilty groups, IncFII-related plasmids possess a great versatility of intracellular adaptation by the rapid evolution of the regulatory sequences of the replicons. Therefore, the backbones of IncFII-related plasmids exhibit a significant heterogeneity in terms of the size and number of replicons [25]. A genetic comparison of two widely distributed *bla*_CTX-M-15_-carrying IncFII-related plasmids pC15-1a and pEK516 revealed three genetic events potentially accounted for all of the differences [26] though a 60-kb region is higher homologous between the two plasmid which is originated from the non-R-determinant region of plasmid R100 [4]. For the 90-kb IncFII-related plasmid identified in this study, it appeared to have genetic components in both the backbone and the resistance region with different origins from those of plasmid R100. In contrast, this 90-kb plasmid appears to be closer to pKF3-94 as both plasmids originated from *K. pneumoniae*, belonged to the IncFII group, carried *bla*_TEM_ and *bla*_CTX-M-15_, and had the same *traF*, *traH*, *traN*, *traU*, *traW* and *trbC* sequences. The close relatedness between the 90-kb plasmid and pKF3-94 was also evidenced by their almost identical EcoRI restriction patterns, suggesting that the two plasmids may contain a common backbone. Interestingly, this 90-kb plasmid was not detected to harbor certain resistance genes such as *bla*_OXA-1_, *qnrB*, *qnrS*, and *aac(6′)-Ib,* which were usually found in other IncFII-related plasmid carrying *bla*_CTX-M-15._ The phenomenon is also found in the resistance region of pKF3-94 as only the IS*Ecp1-bla*_CTX-M-15_ genetic stucture (positions 88110-88985) and *bla*_TEM-1_ (positions 89767-90627) but no IS*26* found on pKF3-94.

IncFII-related plasmids are complex and sophisticated vehicles mediating the dissemination of antimicrobial resistance genes. IncFII-related plasmids may co-exist with other Inc type plasmids and even are able to co-exist each other due to the differences on the *inc* sequence, which controls incompatibility in the same hosts [27]. The co-existence of plasmids were evidenced that three different sizes of plasmids existed in 9 out of 30 transconjugants containing 90-kb IncFII plasmid. The extensive recombination between different plasmids or plasmid fusions could facilitate IncFII-related plasmids to acquire various resistance genes and therefore generate new vehicles encoding multiple resistances. It could be predicted that the local 90-kb IncFII-related plasmid or pKF3-94 plasmid may evolve into plasmids with new phenotypes by acquiring multiple resistant genes during their further spread.

In summary, this study reveals that a high prevalence of *bla*_CTX-M-15_ genes in *K. pneumoniae* was contributed to a 90-kb IncFII-type plasmid which has different backbone structure with the epidemic plasmid found in Europe and other countries. It looks like that the epidemic IncFII plasmid carrying *bla*_CTX-M-15_ is not restricted in southern China but might have been spread the whole country.

## Supporting Information

Figure S1 **Fingerprints of transconjugant containing three conjugative plasmids.**
(DOC)

Figure S2 **Restriction enzyme fingerprints of 90-kb conjugative plasmid digested by EcoRI.**
(DOC)

Figure S3 **Restriction enzyme fingerprints of 90-kb conjugative plasmid digested by EcoRI+HindIII.**
(DOC)

Figure S4 **PFGE patterns of 47 isolates of** ***K. pneumonia.***
(DOC)

Table S1 **Antimicrobial susceptibility of** ***K.pneumoniae*** **isolates carrying** ***bla*** **_CTX-M-15_.**
(DOC)

Table S2 **Primers used in the PCR-based replicon typing scheme.**
(DOC)

Table S3 **Comparison the length of restriction between the 90-kb plasmid and pKF3-94.**
(DOC)

Table S4 **Distribution of the CTX-M genotype in test strains.**
(DOC)
