# Supplementary material for: Epidemic Plasmid Carrying bla CTX-M-15 in Klebsiella penumoniae in China
Source: PLoS One. 2013 Jan 29;8(1):e52222. doi: 10.1371/journal.pone.0052222 (PMC3558504; doi:10.1371/journal.pone.0052222)
Supplement: Figure S1 — Fingerprints of transconjugant containing three conjugative plasmids. (DOC) [file pone.0052222.s001.doc]

1 2 3 4 5


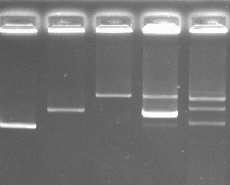


**Figure S1. Fingerprints of transconjugant containing three conjugative plasmids**

Lane1-4: plasmid Marker: lane 1,V517 (sizes: 54, 5.6, 5.1, 3.9, 3.0, 2.7, and 2.1 kb); lane 2,R1 (92 kb); lane 3,R27 (182 kb); lane 4.PLAC (152 kb). Lane 5: the transconjugant containing three different size of plasmid (60-kb, 90-kb, 180-kb)
