# Supplementary material for: Epidemic Plasmid Carrying bla CTX-M-15 in Klebsiella penumoniae in China
Source: PLoS One. 2013 Jan 29;8(1):e52222. doi: 10.1371/journal.pone.0052222 (PMC3558504; doi:10.1371/journal.pone.0052222)
Supplement: Figure S2 — Restriction enzyme fingerprints of 90-kb conjugative plasmid digested by EcoRI. (DOC) [file pone.0052222.s002.doc]

1 2 3 4 5 6 7 M


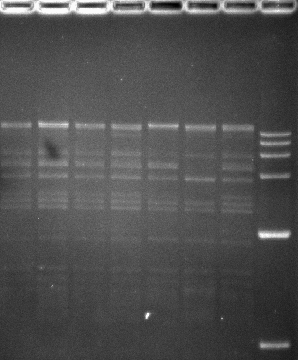


Figure S2. Restriction enzyme fingerprints of 90-kb conjugative plasmid digested by EcoRI.

From lane 1 to lane 7: a single conjugative plasmid carrying blaCTX-M-15;

M: DNA ladder Marker: 10000, 8000,6000, 4000,2000, 500bp.
