# Supplementary material for: Epidemic Plasmid Carrying bla CTX-M-15 in Klebsiella penumoniae in China
Source: PLoS One. 2013 Jan 29;8(1):e52222. doi: 10.1371/journal.pone.0052222 (PMC3558504; doi:10.1371/journal.pone.0052222)
Supplement: Figure S3 — Restriction enzyme fingerprints of 90-kb conjugative plasmid digested by EcoRI+HindIII. (DOC) [file pone.0052222.s003.doc]

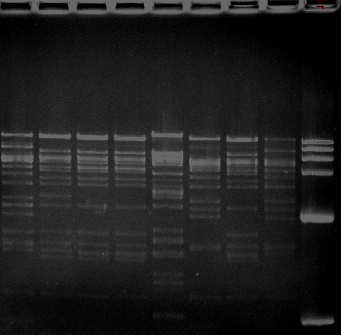
1 2 3 4 5 6 7 8 M

**Fig S3.** Restriction enzyme fingerprints of the 90kb conjugative plasmid

digested by EcoRI+HindIII. Lane 1-lane 8: 90kb conjugative plasmid. M: DNA ladder Marker: 10000, 8000,6000, 4000,2000, 500bp.
