# Supplementary figures and images for: Epidemic Plasmid Carrying bla CTX-M-15 in Klebsiella penumoniae in China
Source: PLoS One. 2013 Jan 29;8(1):e52222. doi: 10.1371/journal.pone.0052222 (PMC3558504; doi:10.1371/journal.pone.0052222)

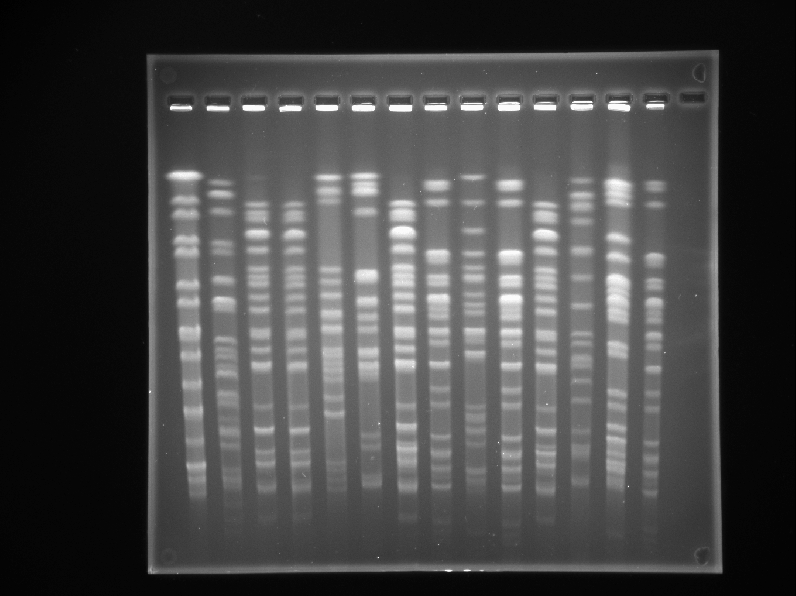


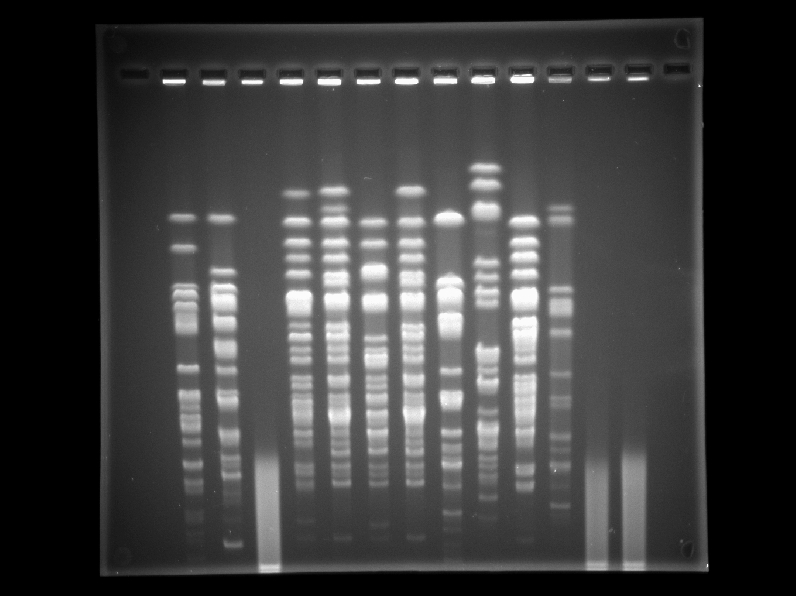


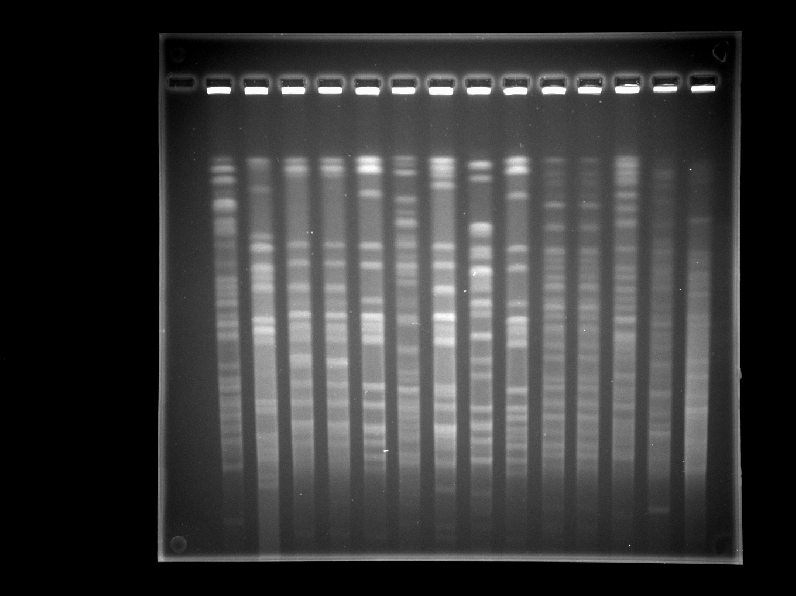


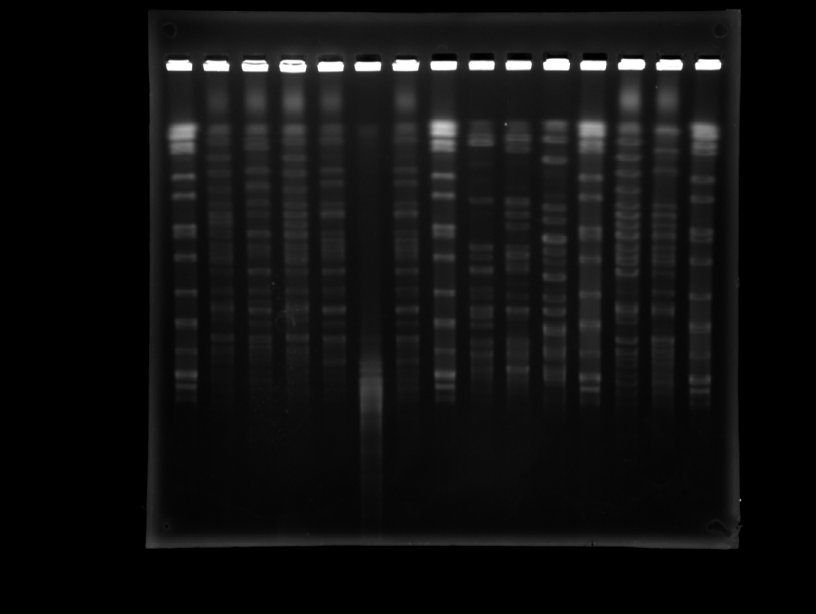


Fig S4. PFGE patterns of 47 isolates of ***K. pneumoniae***

Supplement: Figure S4 — PFGE patterns of 47 isolates of K. pneumonia. (DOC) [file pone.0052222.s004.doc]
