# Supplementary material for: Epidemic Plasmid Carrying bla CTX-M-15 in Klebsiella penumoniae in China
Source: PLoS One. 2013 Jan 29;8(1):e52222. doi: 10.1371/journal.pone.0052222 (PMC3558504; doi:10.1371/journal.pone.0052222)
Supplement: Table S1 — Antimicrobial susceptibility of K.pneumoniae isolates carrying bla CTX-M-15. (DOC) [file pone.0052222.s005.doc]

| Antibiotic name | ***K.****pneumoniae* isolates carrying *bla*_CTX-M-15_ (n=47)_）（）_ | | | | |
| --- | --- | --- | --- | --- | --- |
|  | MIC50 | MIC90 | MIC range  13.8  25.0  17.5  92.2  83.0  42.2  68.1  83.9  61.1  57.7  99.6  92.6  34.3 | R | S |
| Ampicillin | 128 | ≥256 | 8- ≥256 | 89.4 | 2.1 |
| Ampicillin/Sulbactam | 16 | ≥256 | 2- ≥256 | 48.9 | 42.6 |
| Piperacillin | 64 | ≥256 | 8- ≥256 | 89.4 | 4.2 |
| Piperacillin/Tazobactam | 8 | 128 | 1- ≥256 | 10.6 | 76.6 |
| Cefoxitin | 8 | 128 | 2- ≥256 | 40.4 | 57.4 |
| Cefotaxime | 16 | ≥256 | 4- ≥256 | 72.3 | 19.1 |
| Ceftazidime | 16 | ≥256 | 2- ≥256 | 57.4 | 21.3 |
| *Cefoperazone/Sulbactam | 2 | 64 | ≤0.5-128 | 14.9 | 61.7 |
| Cefepime | 8 | 64 | 2- 128 | 68.1 | 19.1 |
| Aztreonam | 64 | ≥256 | 4- ≥256 | 85.1 | 10.6 |
| Imipenem | 0.5 | 4 | ≤0.5- 16 | 2.1 | 97.9 |
| Amikacin | 8 | 128 | 2- 128 | 14.9 | 76.6 |
| Ciprofloxacin | 1 | 16 | ≤0.5- 64 | 29.8 | 59.6 |

Table S1. Antimicrobial susceptibility of ***K.****pneumoniae* isolates carrying *bla*_CTX-M-15_

*: the breakpoint referred to cefoperazone.
