# Supplementary material for: Epidemic Plasmid Carrying bla CTX-M-15 in Klebsiella penumoniae in China
Source: PLoS One. 2013 Jan 29;8(1):e52222. doi: 10.1371/journal.pone.0052222 (PMC3558504; doi:10.1371/journal.pone.0052222)
Supplement: Table S2 — Primers used in the PCR-based replicon typing scheme. (DOC) [file pone.0052222.s006.doc]

**Table S2. Primers used in the PCR-based replicon typing scheme**

| Name |  | DNA sequence | Target site | accession no. | Amplicon size (bp) |
| --- | --- | --- | --- | --- | --- |
| HI1 | FW | 5'-ggagcgatggattacttcagtac-3' | parA-parB | AF250878 | 471 |
|  | RV | 5'-tgccgtttcacctcgtgagta-3' |  |  |  |
| HI2 | FW | 5'-tttctcctgagtcacctgttaacac-3' | iterons | BX664015 | 644 |
|  | RV | 5'-ggctcactaccgttgtcatcct-3' |  |  |  |
| I1 | FW | 5'-cgaaagccggacggcagaa-3' | RNAI | M20413 | 139 |
|  | RV | 5'-tcgtcgttccgccaagttcgt-3' |  |  |  |
| X | FW | 5'-aaccttagaggctatttaagttgctgat-3' | orig | Y00768 | 376 |
|  | RV | 5'-tgagagtcaatttttatctcatgttttagc-3' |  |  |  |
| L/M | FW | 5'-ggatgaaaactatcagcatctgaag-3' | repA,B,C | U27345 | 785 |
|  | RV | 5'-ctgcaggggcgattctttagg-3' |  |  |  |
| N | FW | 5'-gtctaacgagcttaccgaag-3' | repA | NC_003292 | 559 |
|  | RV | 5'-gtttcaactctgccaagttc-3' |  |  |  |
| FIA | FW | 5'-ccatgctggttctagagaaggtg-3' | iterons | J01724 | 462 |
|  | RV | 5'-gtatatccttactggcttccgcag-3' |  |  |  |
| FIB | FW | 5'-ggagttctgacacacgattttctg-3' | repA | M26308 | 702 |
|  | RV | 5'-ctcccgtcgcttcagggcatt-3' |  |  |  |
| W | FW | 5'-cctaagaacaacaaagcccccg-3' | repA | U12441 | 242 |
|  | RV | 5'-ggtgcgcggcatagaaccgt-3' |  |  |  |
| Y | FW | 5'-aattcaaacaacactgtgcagcctg-3' | repA | K02380 | 765 |
|  | RV | 5'-gcgagaatggacgattacaaaacttt-3' |  |  |  |
| P | FW | 5'-ctatggccctgcaaacgcgccagaaa-3' | iterons | M20134 | 534 |
|  | RV | 5'-tcacgcgccagggcgcagcc-3' |  |  |  |
| FIC | FW | 5'-gtgaactggcagatgaggaagg-3' | repA2 | AH003523 | 262 |
|  | RV | 5'-ttctcctcgtcgccaaactagat-3' |  |  |  |
| A/C | FW | 5'-gagaaccaaagacaaagacctgga-3' | repA | X73674 | 465 |
|  | RV | 5'-acgacaaacctgaattgcctcctt-3' |  |  |  |
| T | FW | 5'-ttggcctgtttgtgcctaaaccat-3' | repA | K00053 | 750 |
|  | RV | 5'-cgttgattacacttagctttggac-3' |  |  |  |
| FIIS | FW | 5'-ctgtcgtaagctgatggc-3' | repA | AE006471 | 270 |
|  | RV | 5'-ctctgccacaaacttcagc-3' |  |  |  |
| FrepB | FW | 5'-tgatcgtttaaggaattttg-3' | RNAI/repA | AY234375 | 270 |
|  | RV | 5'-gaagatcagtcacaccatcc-3' |  |  |  |
| K/B | FW | 5'-gcggtccggaaagccagaaaac-3' | RNAI | M93063 | 160 |
| K | RV | 5'-tctttcacgagcccgccaaa-3' |  |  |  |
| B/O | RV | 5'-tctgcgttccgccaagttcga-3' | RNAI | M28718 | 159 |
