# Supplementary material for: Epidemic Plasmid Carrying bla CTX-M-15 in Klebsiella penumoniae in China
Source: PLoS One. 2013 Jan 29;8(1):e52222. doi: 10.1371/journal.pone.0052222 (PMC3558504; doi:10.1371/journal.pone.0052222)
Supplement: Table S3 — Comparison the length of restriction between the 90-kb plasmid and pKF3-94. (DOC) [file pone.0052222.s007.doc]

**Table S3. Comparison the length of restriction between the 90-kb plasmid and pKF3-94**

| pKF3-94 | | | 90-kb plasmid | | |  | |
| --- | --- | --- | --- | --- | --- | --- | --- |
| Ends | **Coordinates** | **Length (bp)** | | Speculated length of the restriction fingerprint in Fig 3. | |  | |
| EcoRI-EcoRI | 86770-7243 | 14693 | | 15000 | |  | |
| EcoRI-EcoRI | 32282-41751 | 9470 | | 9500 | |  | |
| EcoRI-EcoRI | 58365-65435 | 7071 | |  | |  | |
| EcoRI-EcoRI | 74368-81163 | 6796 | |  | |  | |
| EcoRI-EcoRI | 25820-32281 | 6462 | |  | |  | |
| EcoRI-EcoRI | 14740-20745 | 6006 | |  | |  | |
| EcoRI-EcoRI | 49291-55161 | 5871 | | 5900 | |  | |
| EcoRI-EcoRI | 81164-86769 | 5606 | |  | |  | |
| EcoRI-EcoRI | 65436-70543 | 5108 | | 5000 | |  | |
| EcoRI-EcoRI | 7244-11949 | 4706 | | 4700 | |  | |
| EcoRI-EcoRI | 45629-49290 | 3662 | | 3500 | |  | |
| EcoRI-EcoRI | 55162-58054 | 2893 | | 2800 | |  | |
| EcoRI-EcoRI | 23161-25819 | 2659 | | 2600 | |  | |
| EcoRI-EcoRI | 20746-23160 | 2415 | |  | |  | |
| EcoRI-EcoRI | 41752-43886 | 2135 | | 2100 | |  | |
| EcoRI-EcoRI | 72405-74367 | 1963 | |  | |  | |
| EcoRI-EcoRI | 70544-72404 | 1861 | | 1900 | |  | |
| EcoRI-EcoRI | 12923-14739 | 1817 | | 1800 | |  | |
| EcoRI-EcoRI | 43887-45628 | 1742 | | 1700 | |  | |
| EcoRI-EcoRI | 11950-12922 | 973 | | 1000 | |  | |
| EcoRI-EcoRI | 58055-58364 | 310 | |  |  | |  |
