# Supplementary material for: Epidemic Plasmid Carrying bla CTX-M-15 in Klebsiella penumoniae in China
Source: PLoS One. 2013 Jan 29;8(1):e52222. doi: 10.1371/journal.pone.0052222 (PMC3558504; doi:10.1371/journal.pone.0052222)
Supplement: Table S4 — Distribution of the CTX-M genotype in test strains. (DOC) [file pone.0052222.s008.doc]

Table S4. Distribution of the CTX-M genotype in test strains

| CTX-M genotype | NO. of strains (%) |
| --- | --- |
| CTX-M Total | 125（69.1） |
| CTX-M-1 groups | 65（52.0） |
| **CTX-**M-15 | 47（72.3） |
| **CTX-**M-3 | 18（26.7） |
| **CTX-**M-55 | 0（0） |
| **CTX-**M-9 groups | 60（48.0） |
| **CTX-**M-14 | 51（85.0） |
| **CTX-**M-19 | 0（0） |
| **CTX-**M-24 | 4（6.7） |
| **CTX-**M-27 | 1（1.7） |
| **CTX-**M-38 | 2（3.3） |
| **CTX-**M-65 | 1（1.7） |
| **CTX-**M-9 | 1（1.7） |
| M-2 groups、M-8 groups、M-25 groups | 0（0） |
